# Supplementary material for: R-Spondin chromosome rearrangements drive Wnt-dependent tumour initiation and maintenance in the intestine
Source: Nat Commun. 2017 Jul 11;8:15945. doi: 10.1038/ncomms15945 (PMC5508203; doi:10.1038/ncomms15945)
Supplement: Supplementary Information [file ncomms15945-s1.pdf]

Title of file for HTML: Supplementary Information
Description: Supplementary Figures and Supplementary Table

Title of file for HTML: Supplementary Data 1
Description: Gene significantly upregulated in Apc mutant organoids, relative to WT organoids.

Title of file for HTML: Supplementary Data 2
Description: Gene significantly downregulated in Apc mutant organoids, relative to WT organoids.

Title of file for HTML: Supplementary Data 3
Description: Gene significantly upregulated in P-Rspo3 organoids, relative to WT organoids.

Title of file for HTML: Supplementary Data 4
Description: Gene significantly downregulated in P-Rspo3 organoids, relative to WT organoids.

Title of file for HTML: Supplementary Data 5
Description: List of primers and oligonucleotides used.

Title of file for HTML: Supplementary Movie 1
Description: Growth of dox-naïve “WT” organoids cultured in ENR over 72 hours, starting 24 hours following plating in Matrigel. Images were acquired every 1 hour and movies assembled in FIJI (Image J).

Title of file for HTML: Supplementary Movie 2
Description: Growth of dox-naive “WT” organoids cultured in EN over 72 hours, starting 24 hours following plating in Matrigel. Images were acquired every 1 hour and movies assembled in FIJI (Image J).

Title of file for HTML: Supplementary Movie 3
Description: Growth of dox-treated “P-Rspo3” organoids cultured in ENR over 72 hours, starting 24 hours following plating in Matrigel. Images were acquired every 1 hour and movies assembled in FIJI (Image J). Organoids were selected in the absence of RSPO1 for 2 weeks prior to imaging

Title of file for HTML: Supplementary Movie 4
Description: Growth of dox-treated “P-Rspo3” organoids cultured in EN over 72 hours, starting 24 hours following plating in Matrigel. Images were acquired every 1 hour and movies assembled in FIJI (Image J). Organoids were selected in the absence of RSPO1 for 2 weeks prior to imaging.

Title of file for HTML: Peer Review File
Description:

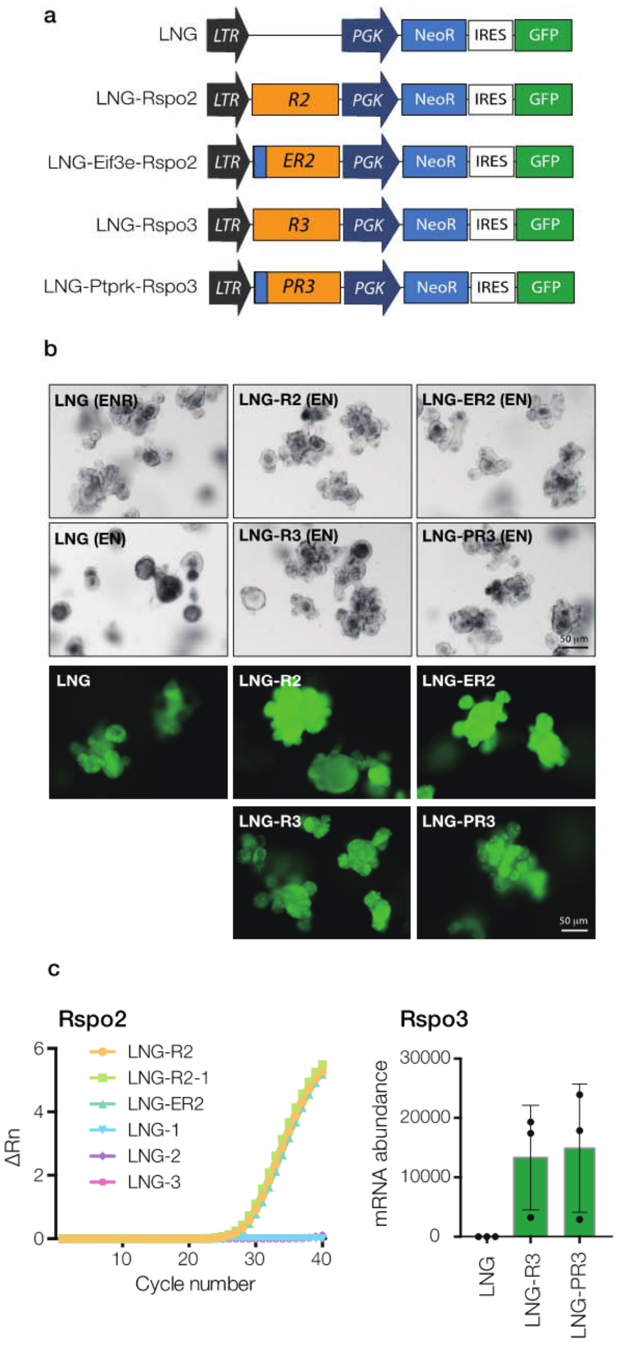

Supplementary Figure 1. Rspo2 and Rspo3 overexpression enables RSPO1-independent organoid growth. **a**. Retroviral vectors used to overexpression Rspo2 and Rspo3 transgenes in mouse organoids **b**. Brightfield and fluorescent images of transduced organoids cultures in ENR or EN, as indicated. **c**. Rspo2 and Rspo3 expression in transduced organoids, measured by q-RT-PCR. Graph represents mean values +/- s.d.. Rspo2 expression is represented as the raw amplification curve because relative expression could not be plotted due to lack of detection in control cultures.

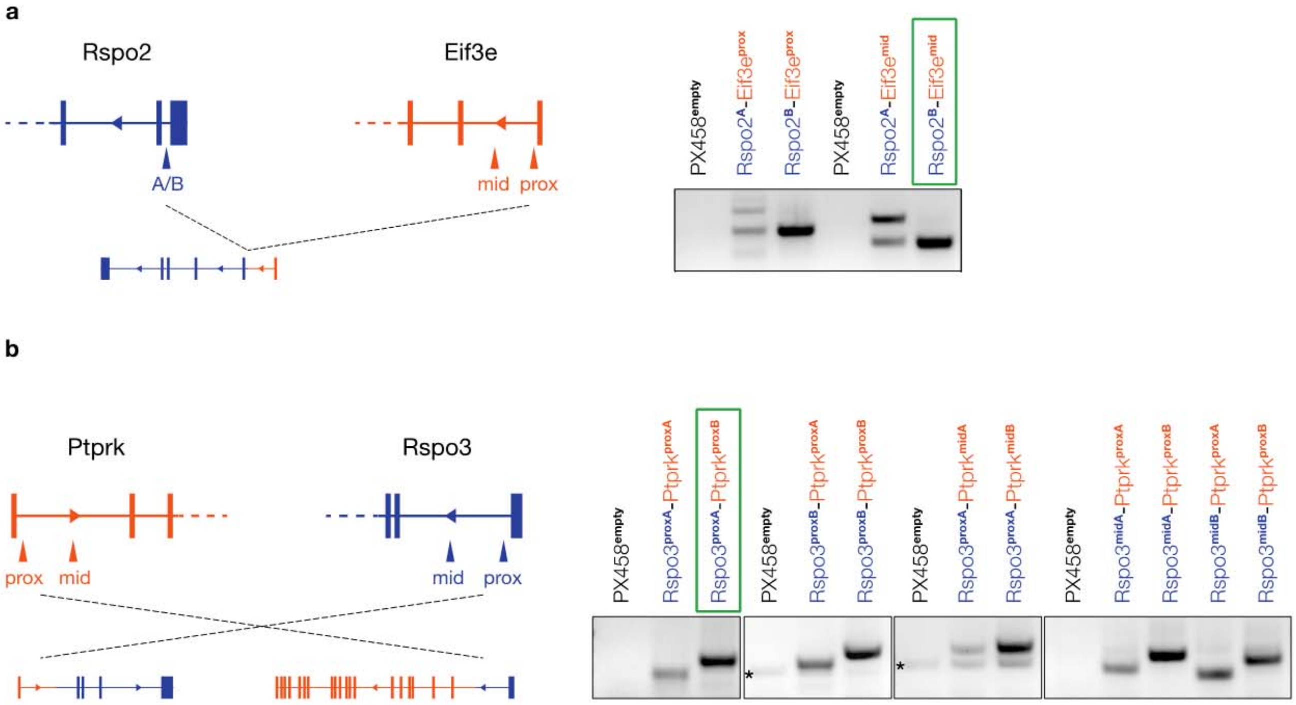

Supplementary Figure 2. Dual sgRNA expression enables Cas9-driven *E-Rspo2* and *P-Rspo3* chromosome rearrangements. Left: Schematic representation of sgRNAs targeting in the introns of Eif3e and Rspo2 (**a**), and Ptprk and Rspo3 (**b**). Right: Fusion-specific PCRs following co-transfection of 3T3s cells with different sgRNA combinations, as indicated. *Non-specific bands also present in WT control DNA. sgRNA combinations used for transgenic mouse production are highlighted by green boxes.

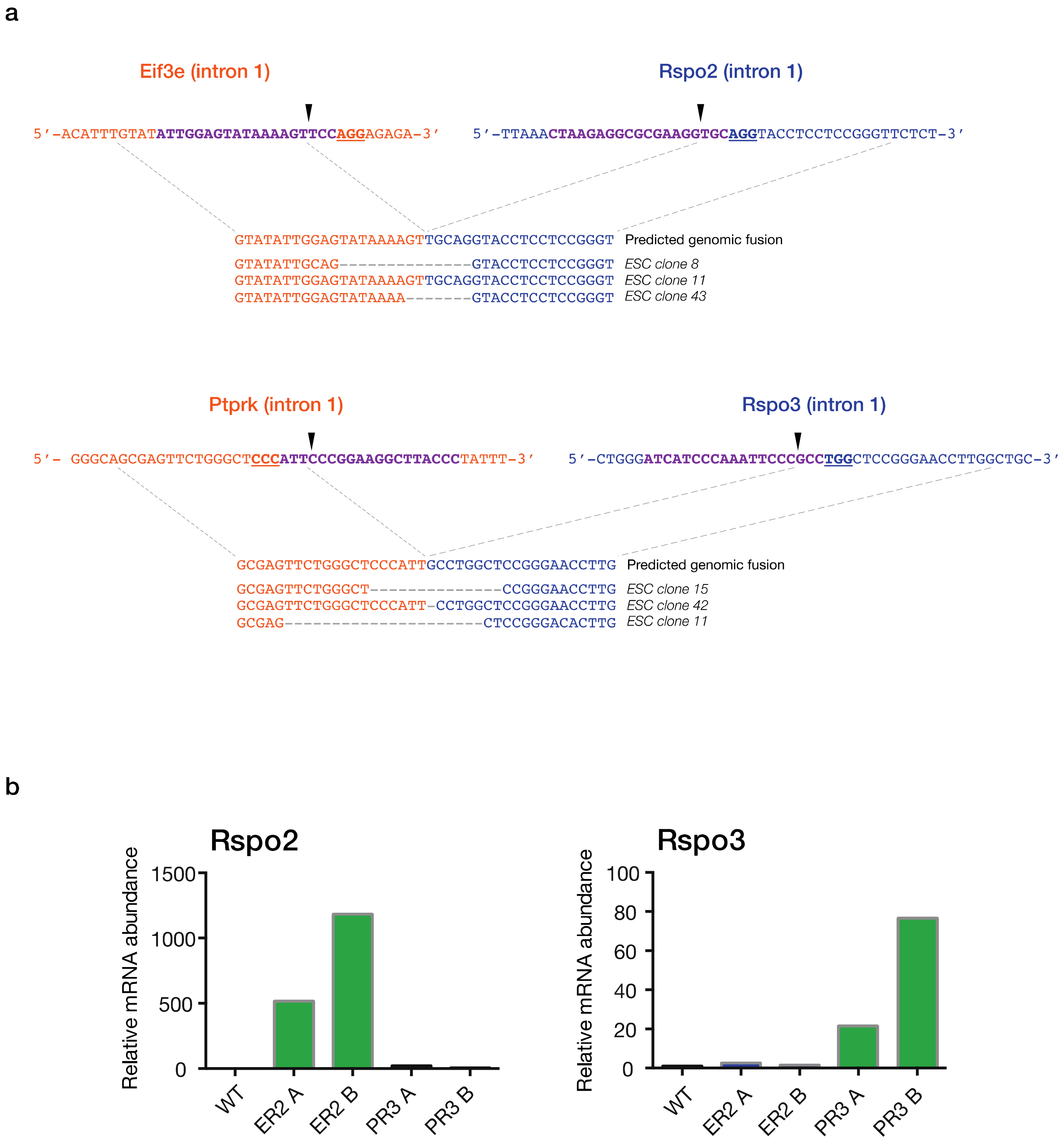

Supplementary Figure 3. Inducible transgenic CRISPR drives genomic rearrangements and overexpression of RSPO genes in ESCs. a. Position of sgRNAs targeting Eif3e, Rspo2, Ptprk, and Rspo3 and corresponding genomic fusions junctions induced in individual dox-treated ESC clones (n=3 analyzed). b. Relative expression of Rspo2 (left) and Rspo3 (right) in ESC clones carrying *E-Rspo2* or *P-Rspo3* rearrangements.

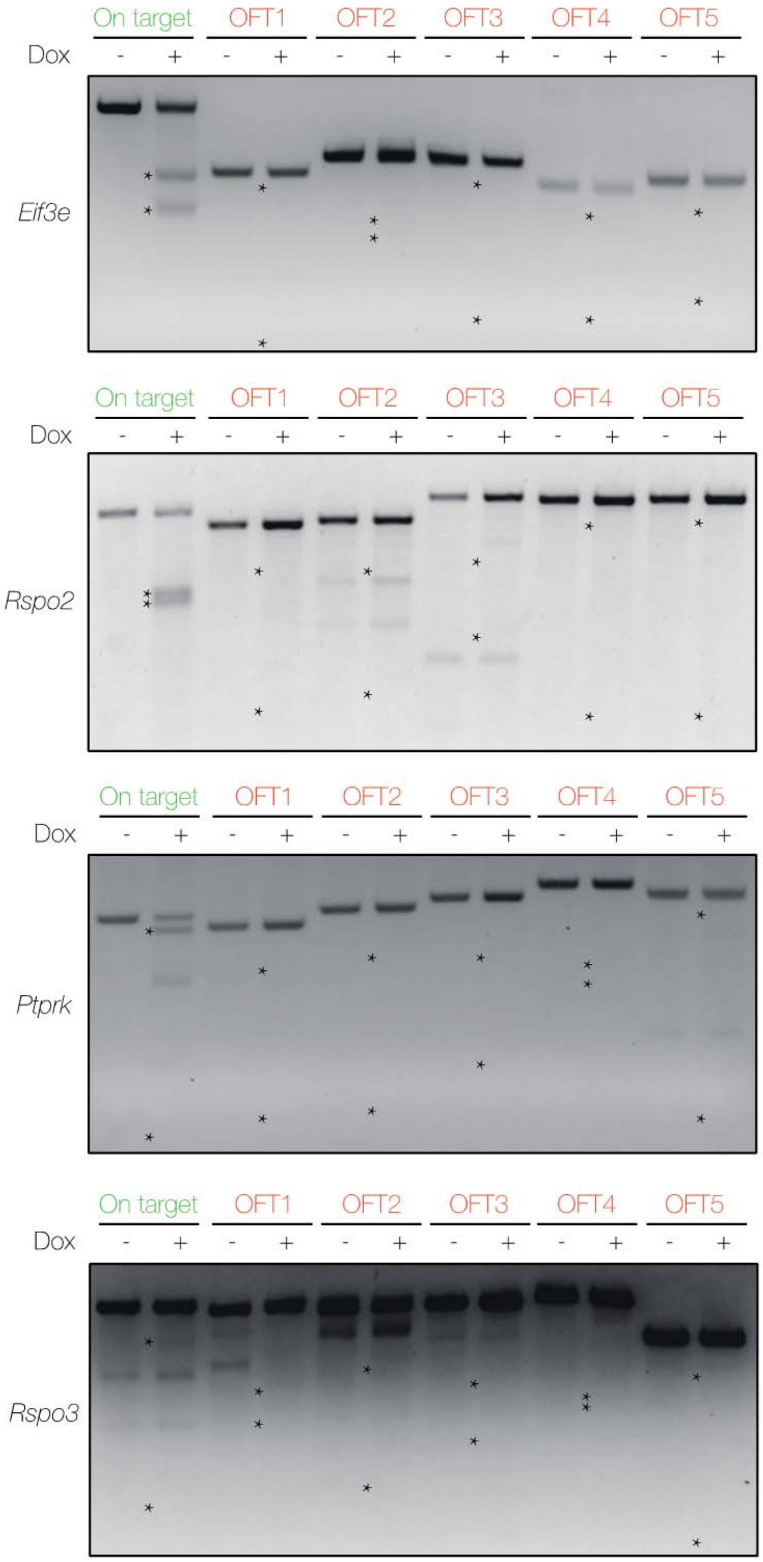

Supplementary Figure 4. Off-target analysis of sgRNAs for creating *E-Rspo2* and *P-Rspo3* rearrangements. T7 assays on naive and dox-treated transgenic ES cells to examine predicted off-target sites of Eif3e, Rspo2, Ptprk and Rspo3 sgRNAs. Predicted sizes of bands released by off-target cleavage are indicated by *.

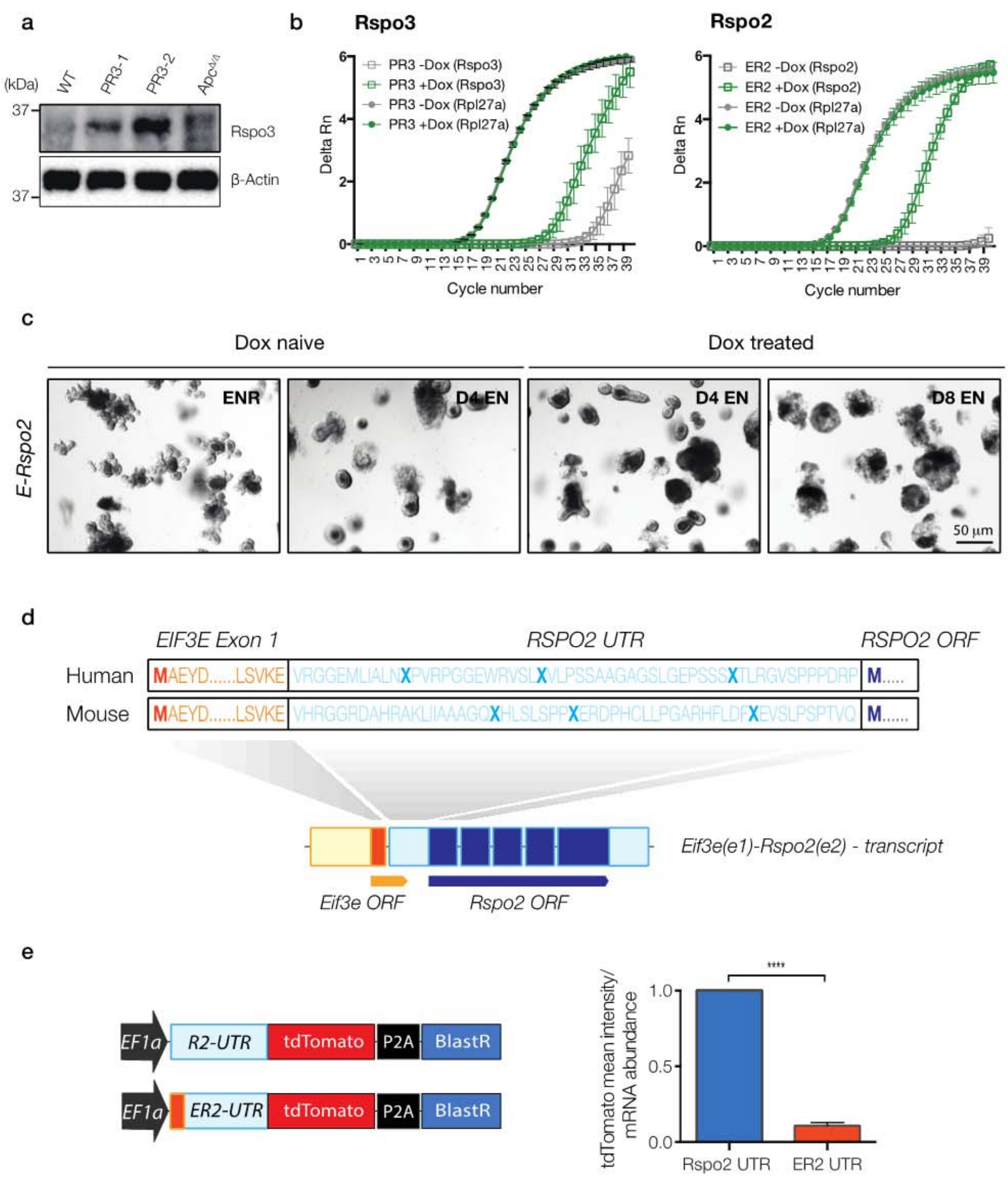

Supplementary Figure 5. Inducible transgenic CRISPR drives genomic rearrangements and overexpression of Rspo genes in intestinal organoids. **a**. Western Blot shown the overexpression of R-spondin3 from the *P-Rspo3* organoids. **b**. Expression of *Rspo2* (right) and *Rspo3* (left) mRNA in polyclonal *R26-rtTA/c3GIC9-E-Rspo2* and *R26-rtTA/c3GIC9-P-Rspo3* intestinal organoid populations following 6 days of dox treatment. Expression is represented as a raw amplification curve due to low/no detection in control cultures. Individual points represent mean of 2 independent organoid cultures +/- s.d. **c**. Brightfield images of R26-rtTA/c3GIC9-E-Rspo2 organoids cultured in ENR or EN, as indicated. Organoids carrying *E-Rspo2* rearrangements do not expand in the absence of exogenous RSPO1. Scale bar, 50 µm. **d**. Schematic representation of *E-Rspo2* fusion in human and mouse. Stop codons in *Rspo2* UTR region are labeled as X. **e**. Structure of tdTomato reporter constructs for testing translational efficiency affected by the *E-Rspo2* UTR (left) and graph represents tdTomato mean fluorescence intensity, relative to mRNA abundance (right). Bars represent mean values +/- s.d. ****p<0.0001, two-sided t-test with Welch correction.

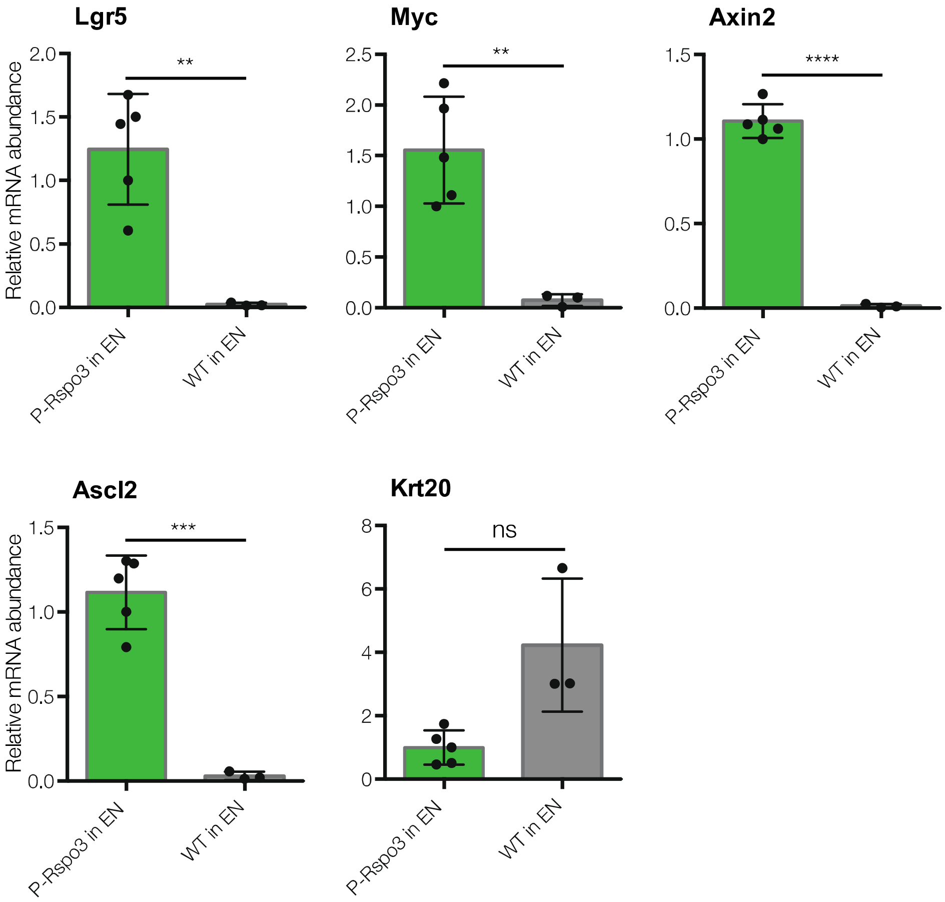

Supplementary Figure 6. Wnt targets and stem cell markers are maintained in *P-Rspo3* organoids following RSPO1 withdrawal. Graphs represent mean mRNA abundance (+/- s.d.) of Wnt target genes (Lgr5, Myc, Axin2 and Ascl2) and differentiation marker (Krt20) from *P-Rspo3* and WT organoids cultured in EN medium for 3 days. n≥3, bars represent mean values +/- s.d., **p<0.01, ***p<0.001, ****p<0.0001, two-sided t-test with Welch correction.

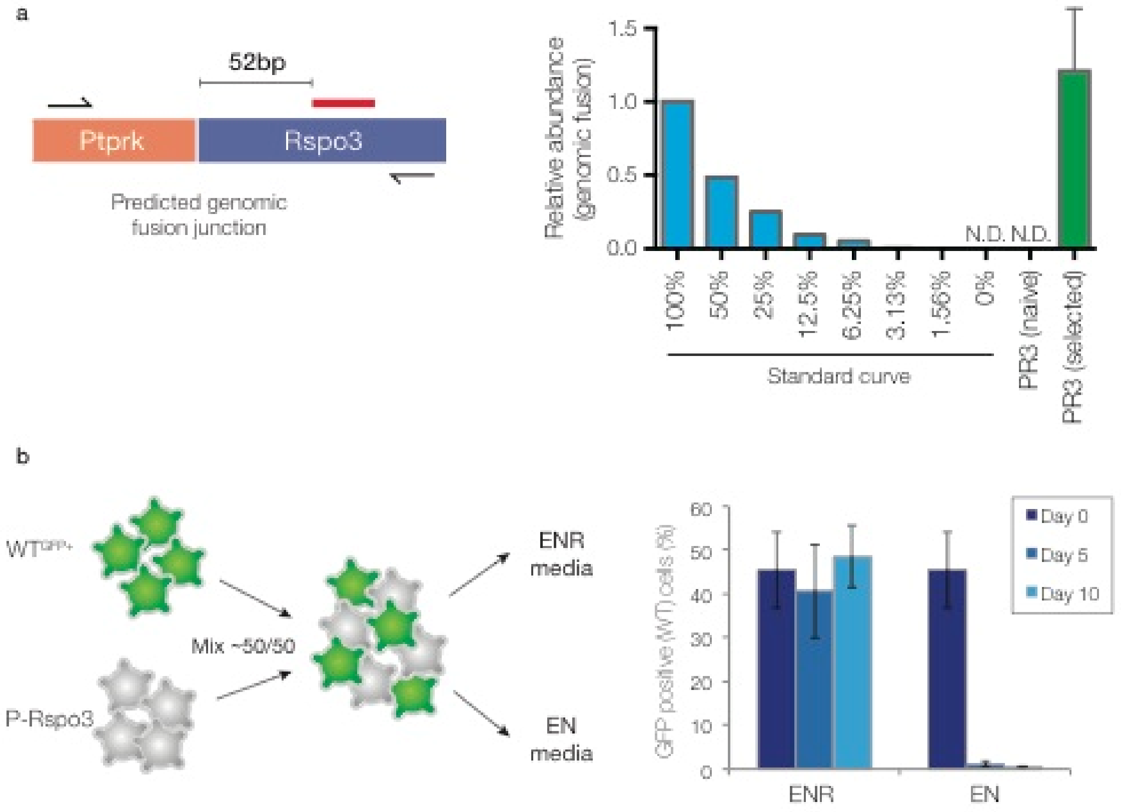

Supplementary Figure 7. The *P-Rspo3* rearrangement in enriched in EN-selected organoid cultures. **a.** *Left*: Schematic representation of the Taqman assay used to quantify the frequency of the *P-Rspo3* inversion in polyclonal cell populations. The probe was positioned in *Rspo3*, as far as possible from the predicted fusion junction, to avoid disrupted probe binding simply due deletions at the junction site. *Right*: Graph shows standard curve generated using isogenic ESC clones by mixing *P-Rspo3* gDNA with *wildtype* gDNA at percentages indicated. Dox-naïve *P-Rspo3* organoids show no evidence of the rearrangement by Taqman assay, whereas dox-treated, EN-selected cultures show a rearrangement frequency of 1.2+/- 0.3 (n=3, bars represent mean values +/- s.d.). This implies that the vast majority of cells in the culture carry the rearrangement. **b**. Schematic representation of co-culture assay. GFP positive (*wildtype*) crypts are mixed with *P-Rspo3* positive crypts at approximately 50:50 ratio, and cultured in the presence (ENR media) or absence of exogenous RSPO1 (EN media). Bar graph shows the percentage of GFP positive cells 0, 5, and 10 days following culture in ENR or EN media. Thus, *P-Rspo3* positive cells cannot support the growth of wildtype (non-Rspo expressing) cells in the absence of exogenous RSPO1 (n=2, bars represent mean value +/- s.d.).

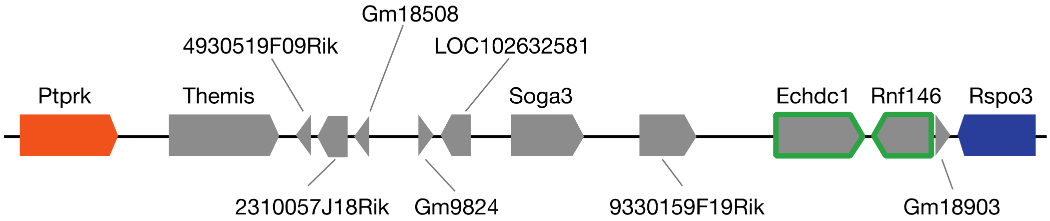

Average RPKM	$Apc^{\Delta/\Delta}$	WT	P-Rspo3
**Ptprk**	21.18	14.49	9.21
Themis	0.00	0.00	0.00
4930519F09Rik	0.02	0.03	0.04
2310057J18Rik	0.00	0.00	0.00
Gm18508			
Gm9824	0.26	0.18	0.21
LOC102632581			
Soga3	0.00	0.00	0.00
9330159F19Rik	0.00	0.00	0.00
Echdc1	33.02	35.94	35.29
Rnf146	23.03	17.03	16.50
Gm18903			
**Rspo3**	0.36	0.00	9.60

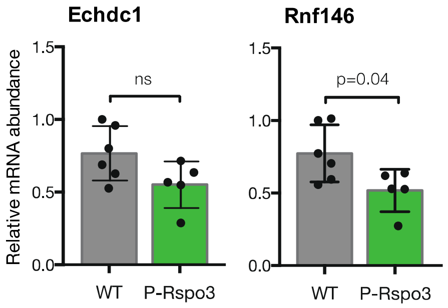

Supplementary Figure 8. The *P-Rspo3* inversion does not disrupt expression of intervening genes. Schematic representation of the genomic *Ptprk* and *Rspo3* genomic locus, highlighting all intervening annotated transcripts. Lower left: Mean RPKM values of each transcript from RNAseq in indicated organoid genotypes. Blank rows indicate the transcript was not identified during RNAseq mapping. Transcripts with RPKM values >1 were validated by q-RT-PCR and showed a minor decrease in expression in P-Rspo3 organoids (bars represent mean values +/- s.d., n>5, two-sided t-test with Welch correction).

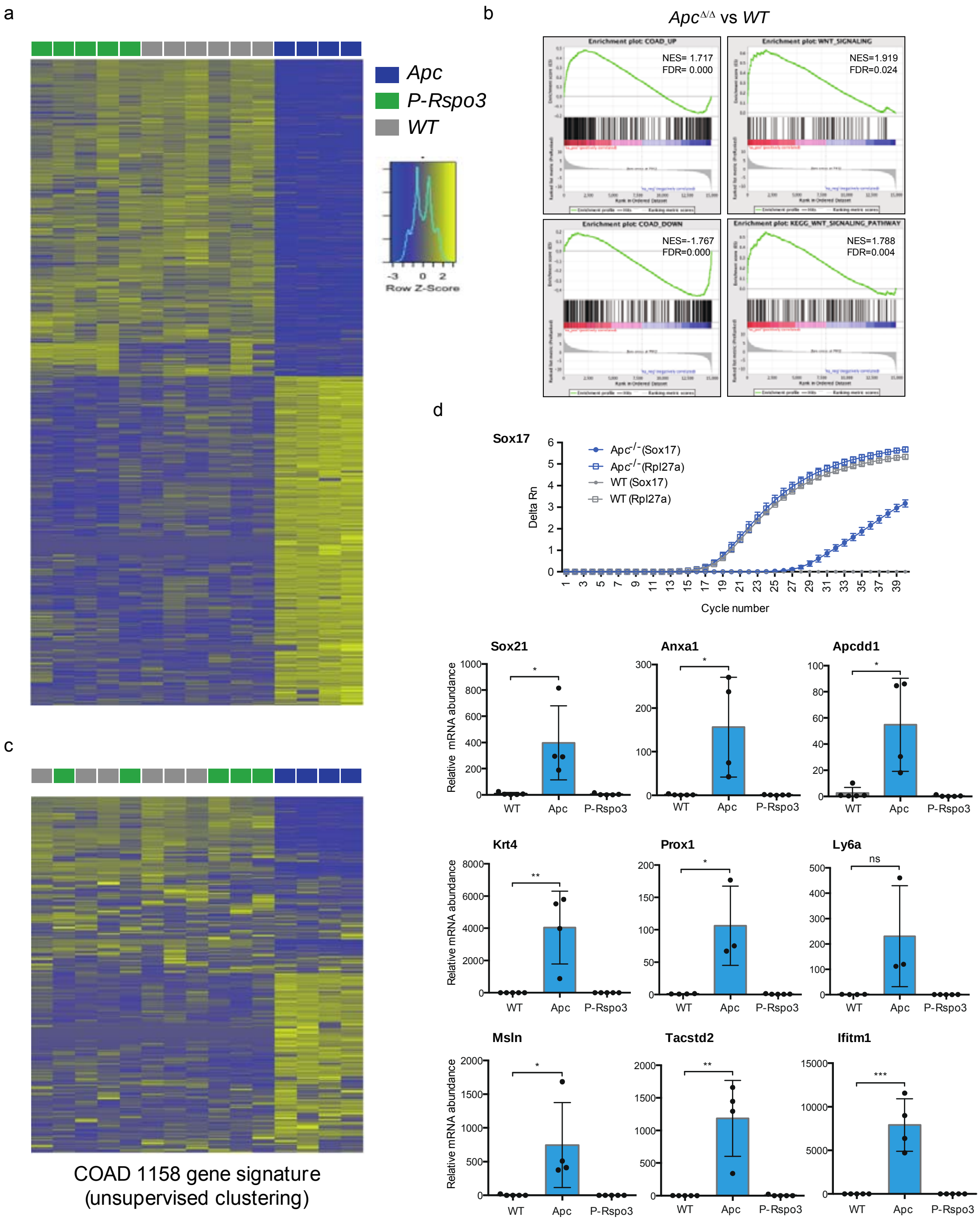

Supplementary Figure 9. Apc disruption drives gene expression changes associated with the Wnt pathway and human colorectal adenocarcinoma. **a**. Heat map showing expression of 5,294 genes with a log2 fold change (log2FC)>1, (adj. p-value<0.05) between *Apc* mutant (“Apc”) and *wildtype* (“WT”) organoids (2598 upregulated and 2696 downregulated). **b**. Gene Set Enrichment Analysis (GSEA) plots showing significant enrichment of gene signatures that define colorectal adenocarcinoma (COAD) and Wnt pathway activation, in Apc mutant organoids, **c**. Heat map from unsupervised clustering of organoid RNAseq data, using a 1158 COAD gene signature developed from the TCGA CRC cohort (see Methods). Apc mutant (blue) organoids cluster together, while P-Rspo3 (green) and WT (grey) samples are mixed. **d**. q-RT-PCR validation of a selection of well-expressed, highly deregulated genes between Apc mutant and WT organoids (Bars represent mean values +/- s.d., n>4 *p<0.05, **p<0.01, ***p<0.001, two-sided t-test with Welch correction).

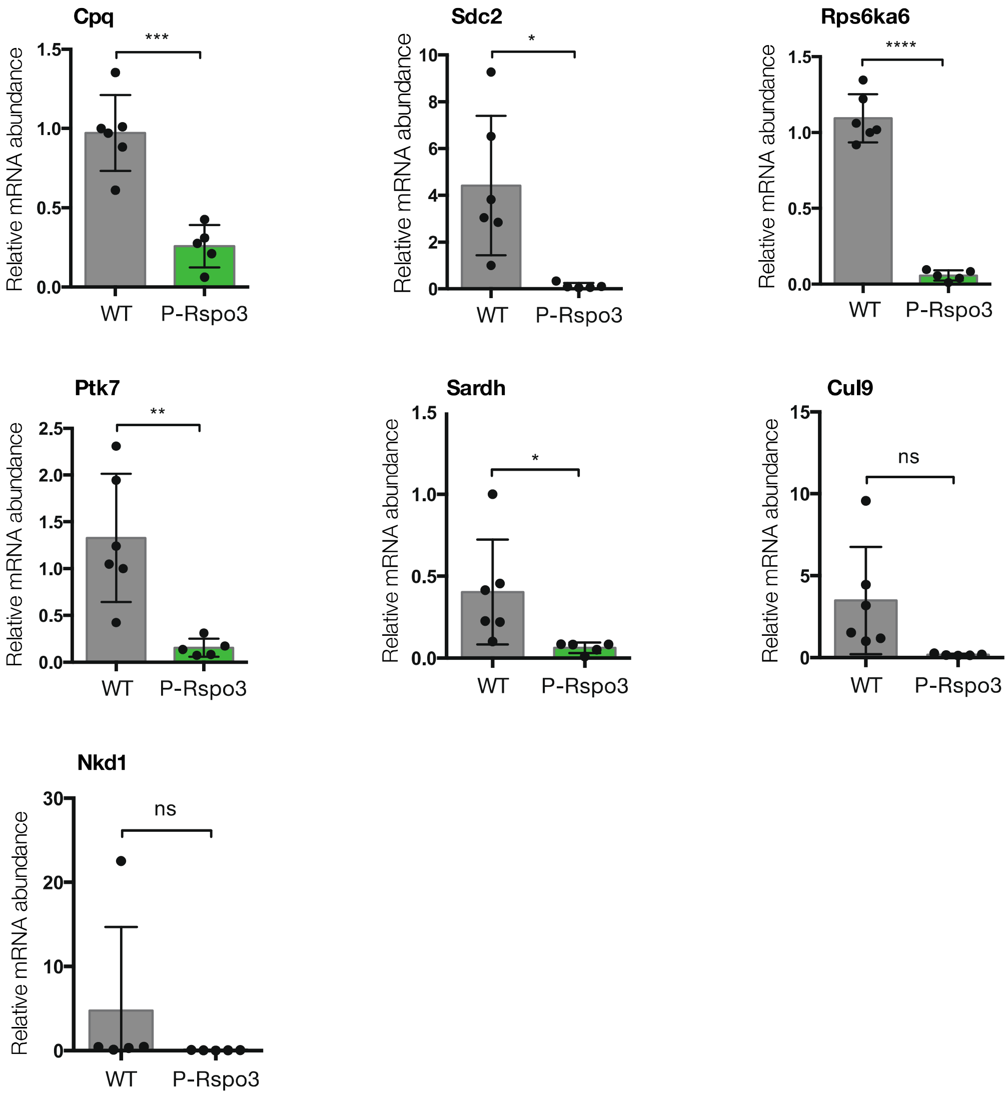

Supplementary Figure 10. The *P-Rspo3* rearrangement drives downregulation of a small subset of genes. q-RT-PCR validation of each of the significantly downregulated genes between *P-Rspo3* rearranged and *WT* organoids (Bars represent mean values +/- s.d., n>4 *p<0.05, **p<0.01, ***p<0.001, two-sided t-test with Welch correction).

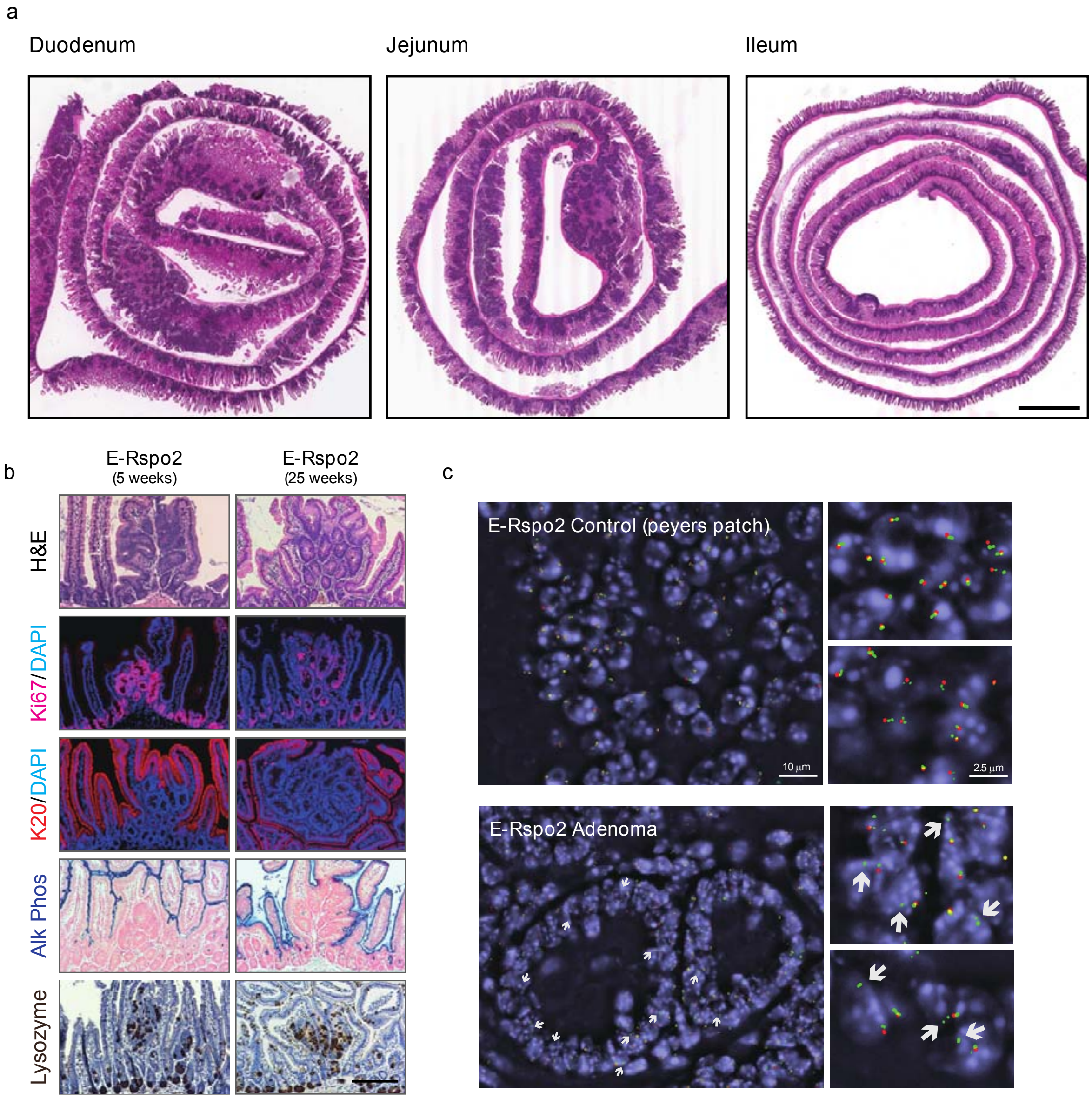

Supplementary Figure 11. *Rspo* rearrangements block differentiation and drive ectopic Paneth cell production in the intestine. **a**. Swiss roll cross-sections of the duodenum, proximal jejunum and ileum, from a representative *R26-rtTA/c3GIC9-P-Rspo3* mouse, 6 weeks post Cas9 induction. Proximal regions of the small intestine show more dramatic histological disruption than distal regions. Scale bar represents 2mm. **b**. Immunohistochemical (H&E, alkaline phosphatase, and Lysozyme), and immunofluorescent (Ki67 and Keratin 20) stains of intestinal sections from *R26-rtTA/c3GIC9-E-Rspo2* animals treated with dox (200mg/kg) for 10 days, and harvested at indicated times. Scale bars, 100µm. **c**. DNA FISH staining on intestinal sections from *R26-rtTA/c3GIC9-E-Rspo2* animals. Immune cells within Peyers patches are shown as a normal control. *E-Rspo2* adenomas are enriched for the E-Rspo2 deletion, highlighted with white arrows.

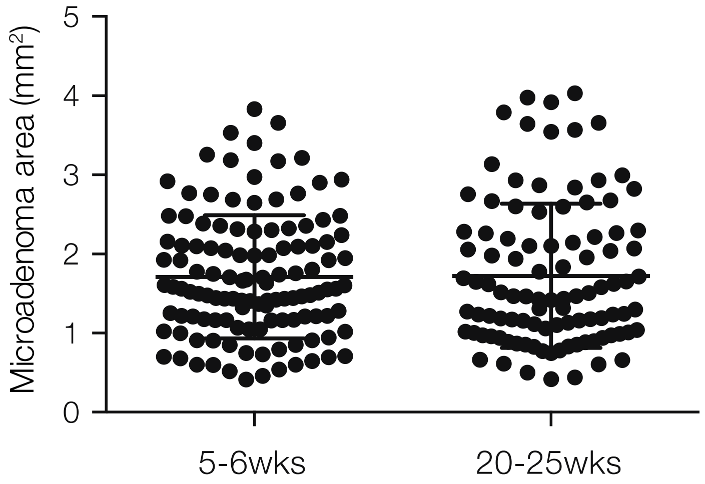

Supplementary Figure 12. Tumors from *R26-rtTA/c3GIC9-E-Rspo2* mice do not progress over time. Dotplot shows area of individual microadenoma in *R26-rtTA/c3GIC9-E-Rspo2* mice 5-6 weeks or 20-25 weeks, post dox treatment. Lines represent mean values +/- s.d.; tumors from 4 independent mice/timepoint are shown.

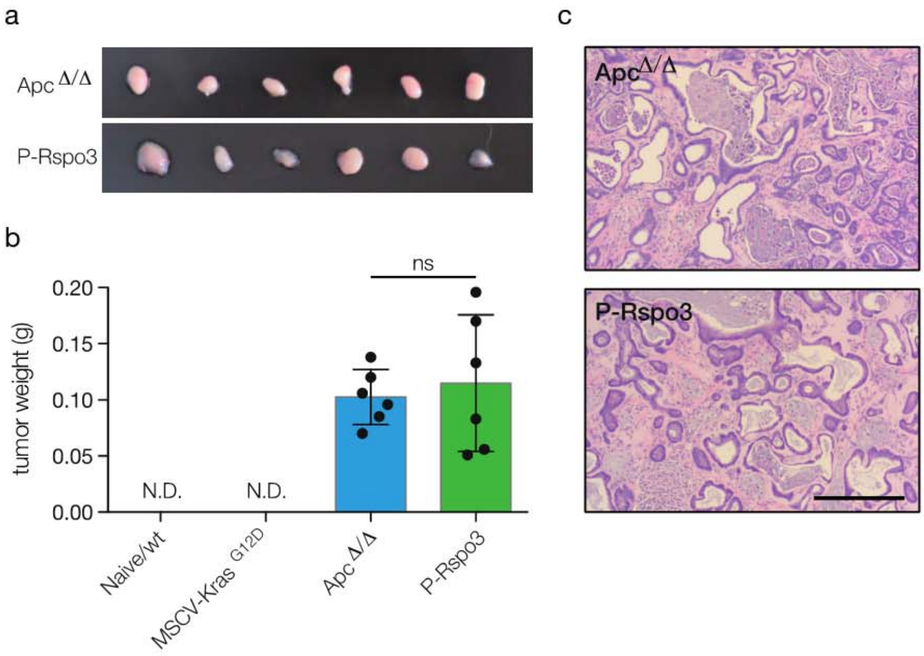

Supplementary Figure 13. Subcutaneous transplantation of *Apc*-deleted organoids and *P-Rspo3* orgnoids. **a**. Images of individual tumors isolated from nude mice transplanted with *Apc*-deleted organoids (upper) and *P-Rspo3* organoids (lower). **b**. Weight of tumors isolated from subcutaneous transplantations (n≥4, Bars represent mean values +/- s.d., two-sided t-test with Welch correction). **c**. H&E stains of *Apc*-deleted (upper) and *P-Rspo3* (lower) subcutaneous tumors, showing equivalent tumor histology. Scale bars, 100µm

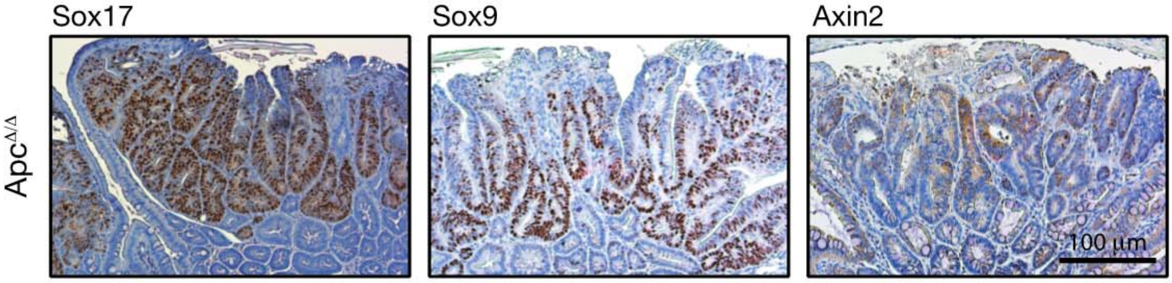

Supplementary Figure 14. Immunohistochemical staining showing upregulation of Sox17, Sox9, and Axin2 in adenomas within the small intestine of 4OHT treated *Lgr5-CreER/Apc*$^{fl/fl}$ animals. Scale bars, 100µm

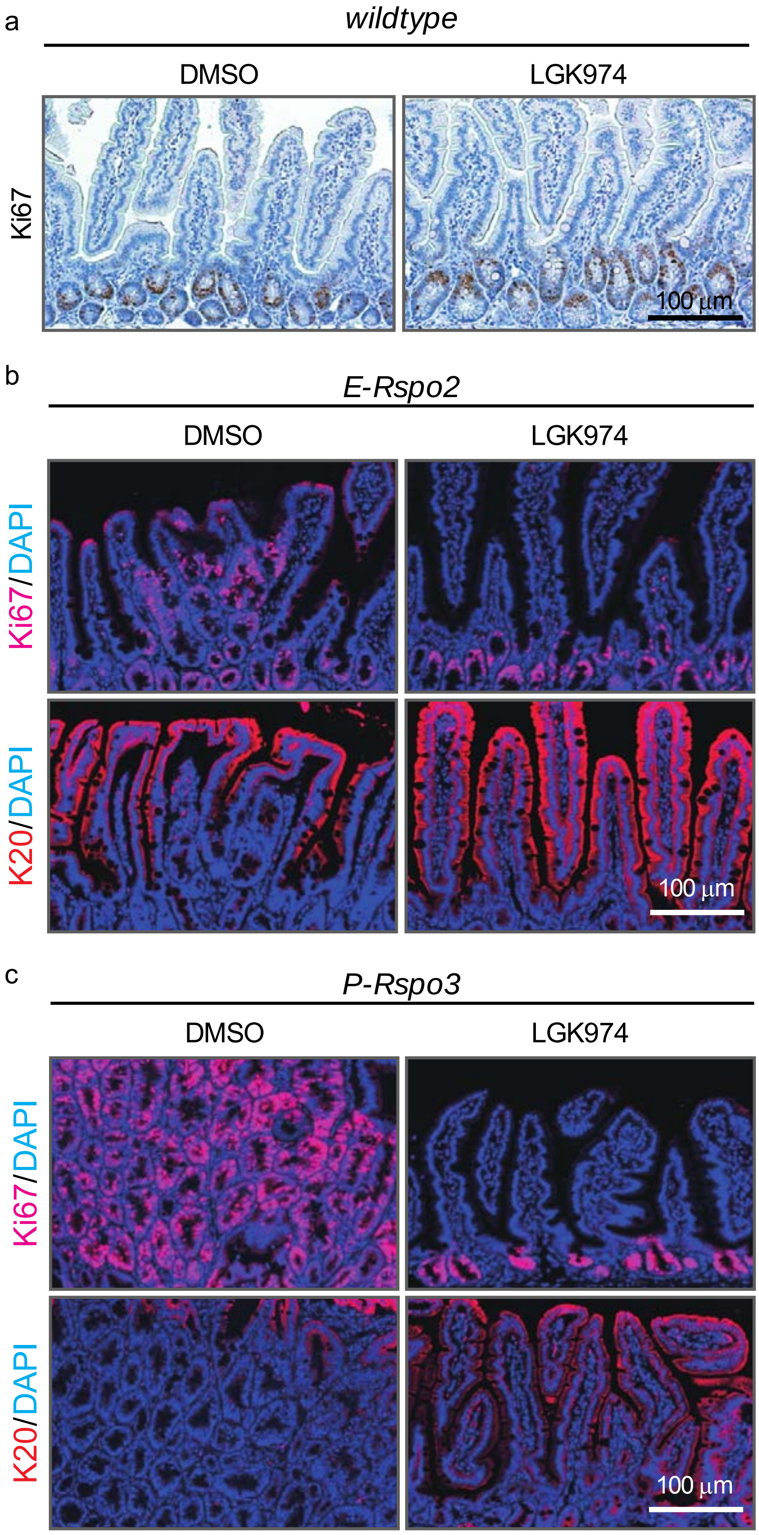

Supplementary Figure 15. Rspo rearranged tumors and organoids are sensitive to Porcn inhibition. Immunohistochemical and immunofluorescent images of intestinal sections from wildtype (**a**), E-Rspo2 (**b**), and P-Rspo3 (**c**) mice treated with either DMSO or LGK974 (5mg/kg, 7 days), showing markers of proliferation (Ki67) and differentiation (K20). One week of LGK974 treatment eliminates hyperproliferative lesions, but does not affect proliferation in intestinal crypts. Scale bars, 100µm.

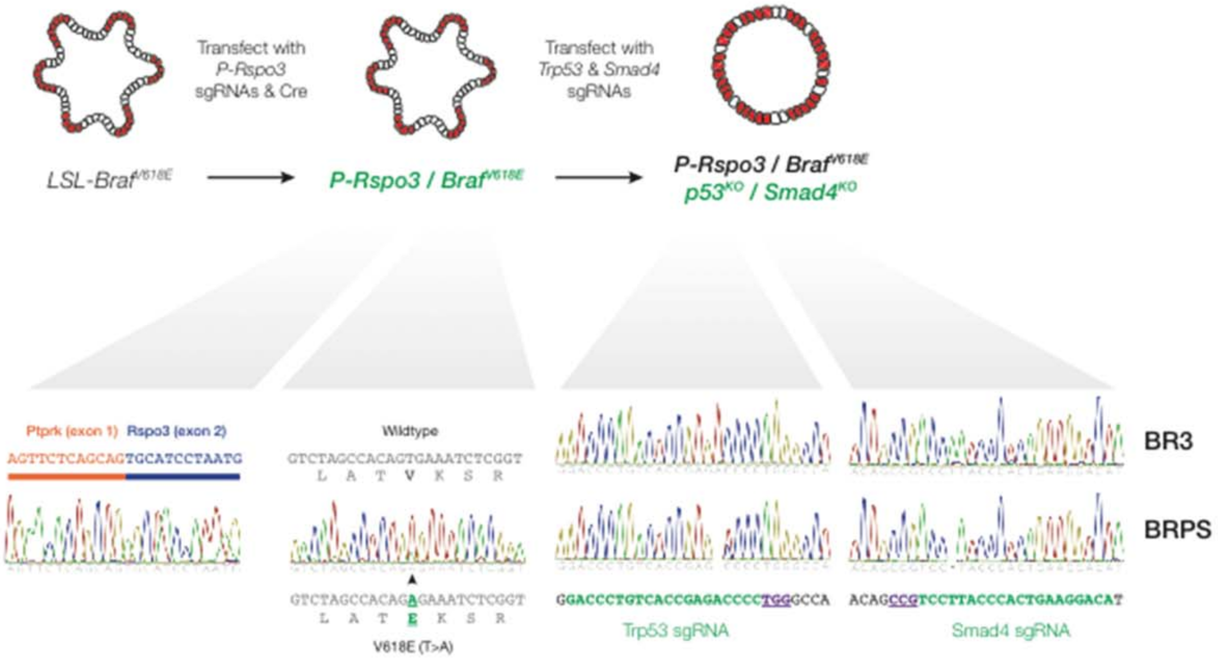

Supplementary Figure 16. Sequential CRISPR-mediated mutagenesis in intestinal organoids. Upper: Schematic representation of the generation of *P-Rspo3, BrafV618E* alterations, and loss of function mutations in *Trp53* and *Smad4*. Lower: Validation of genetic alterations described above, showing Sanger sequencing chromatograms of: the *P-Rspo3* mRNA fusion junction, mRNA expression of the *BrafV618E* (T>A) mutation, and genomic sequences of targeted *Trp53* and *Smad4* loci in BR3 and BRPS organoids.

Uncropped gels in Figure 1

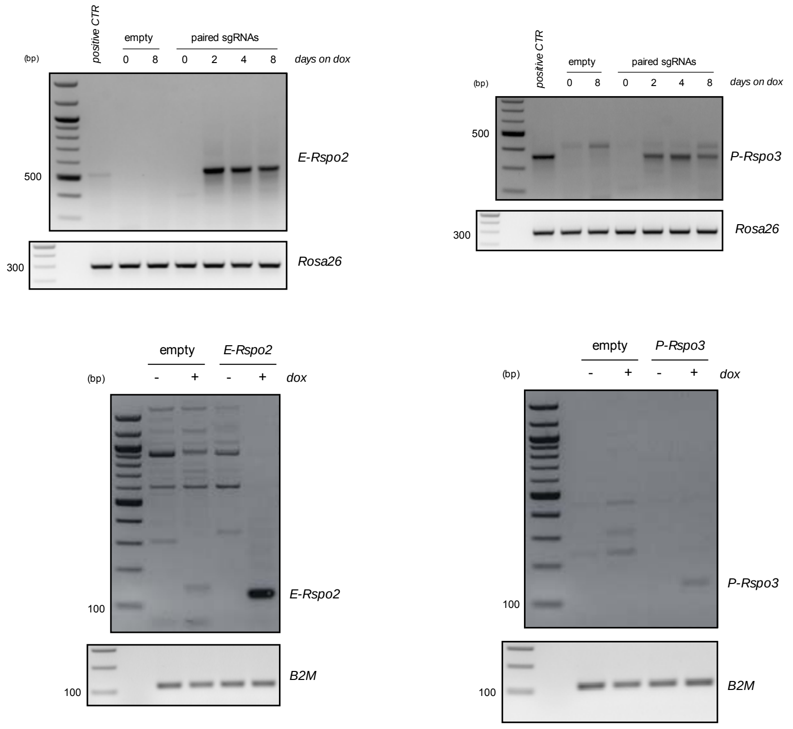

Uncropped gel in Figure 2

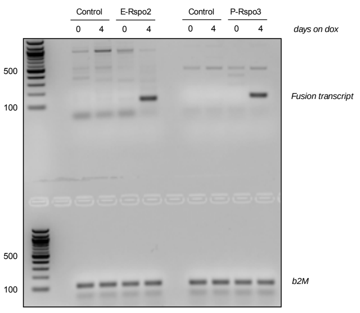

Uncropped gel in Figure 3

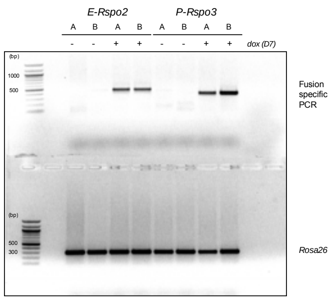

Supplementary Figure 17: Uncropped gels, including markers, from images in Figures 1, 2, and 3.

Supplementary Table 1. Predicted off-target sites for each sgRNA used in iCRISPR mouse models

	Sequence (excl Pos. 1)	Score	Mismatches	UCSC gene	Locus
**Eif3e**	ATTGGAGTATAAAAGTTCCAGG	64			
	ATTGGATGATAAAAGTTCCAGG	1.6	2		chr19:-31140148
	GTTAGAGTAGAAAAGTTCCTGG	1.5	3		chr3:+38476962
	ATGGGACCATAAAAGTTCCCGG	1.5	3		chr11:-78177234
	GTTGAAGTGTAAAAGTTCCTAG	1.5	3		chr1:-77808864
	ATTTAACTATAAAAGTTCCAAG	1.4	3		chr3:+157946956
**Rspo2**	CTAAGAGGCGCGAAGGTGCAGG	92			
	CGCAGCGGCGCGAAGGTGCCAG	0.9	3	NM_146117	chr2:-25145910
	GTAGGAGGCCCGAAGGTGCAAG	0.8	3		chr11:-45642881
	CTGAGAGGGGAGAAGGTGCAGG	0.7	3		chr4:+107139081
	CTGAGAAGGGCCAAGGTGCAGG	0.5	4		chr8:-8405093
	CTAAGAGGAGAGAAGCTGCTAG	0.5	3		chr15:-18323644
**Ptprk**	GGGTAAGCCTTCCGGGAATGGG	91			
	GAGTATGCCATCCGGGAATGGG	1	3		chr4:-106627485
	GGGGAATCTTTCCGGAAATCAG	0.5	4		chrX:+10774848
	GGGAAGGCCTTCCGGGAAAAGG	0.5	3	NM_001080817	chr9:+31154438
	GGAAAAGCCTTCTGGGAATCGG	0.4	3		chr7:+103483395
	GAGTAAGAATTCCGGGAAAAGG	0.4	4		chrX:-88561129
**Rspo3**	ATCATCCCAAATTCCCGCCTGG	93			
	ACGGTCCCAAATTCCCGCCAGG	1.3	3		chr9:-120843209
	AACATCTCAAATTCCAGCCCAG	0.6	3		chr13:-7134334
	GTTACCCCAACTTCCCGCCAAG	0.4	4		chr10:-126684474
	AGCCTCCCAAATCCCCGCCAAG	0.4	3		chr3:-57896983
	TTCTTCCCAAATTCCCCCCAAG	0.3	3		chr2:-94124858
